# Overexpression of heat shock protein 47 is associated with increased proliferation and metastasis in gastric cancer

**DOI:** 10.1186/s44342-024-00010-7

**Published:** 2024-06-17

**Authors:** Jieun Lee, Jung-Ah Hwang, Seung-Hyun Hong, Seon-Young Kim, Donghyeok Seol, Il Ju Choi, Yeon-Su Lee

**Affiliations:** 1 Department of Surgery, Seoul National University Bundang Hospital, Seongnam, Republic of Korea; 2 Genomics Core Facility, Research Core Center, Research Institute, National Cancer Center, Goyang, Republic of Korea; 3 Enzynomics Co. Ltd, Daejeon, Republic of Korea; 4 Center for Gastric Cancer, National Cancer Center, Goyang, Republic of Korea; 5 Rare Cancer Branch, Research Institute, National Cancer Center, Goyang, Republic of Korea

**Keywords:** HSP47, SERPINH1, Proliferation, MMP-7, Promoter methylation

## Abstract

Here, we investigated that the heat shock protein 47 (HSP47) plays a crucial role in the progression of gastric cancer (GC). We analyzed HSP47 gene expression in GC cell lines and patient tissues. The HSP47 mRNA and protein expression levels were significantly higher in GC cell lines and tumor tissues compared to normal gastric mucosa. Using siRNA to silence the expression of HSP47 in GC cells resulted in a significant reduction in their proliferation, wound healing, migration, and invasion capacities. Additionally, we also showed that the mRNA expression of matrix metallopeptidase-7 (MMP-7), a metastasis-promoting gene, was significantly reduced in HSP47 siRNA-transfected GC cells. We confirmed that the HSP47 promoter region was methylated in the SNU-216 GC cell line expressing low levels of HSP47 and in most non-cancerous gastric tissues. It means that the expression of HSP47 is regulated by epigenetic regulatory mechanisms. These findings suggest that targeting HSP47, potentially through its promoter methylation, could be a useful new therapeutic strategy for treating GC.

**Supplementary Information:**

The online version contains supplementary material available at 10.1186/s44342-024-00010-7.

## Introduction

Gastric cancer (GC) is the fifth most common malignancy and the fourth leading cause of cancer-related deaths in the world [1]. In an effort to identify novel genes related to GC progression that can be used to understand GC development, we previously profiled gene expression in human GC and adjacent normal tissues [2]. One gene, serpin peptidase inhibitor, clade H, member 1 (*SERPINH1*), the gene encoding HSP47, was upregulated in gastric tumor tissues.

HSP47 belongs to the serine protease inhibitor (serpin) superfamily, but has no protease inhibitor activity [3]. It was originally discovered as a cell surface collagen-binding protein, colligin, and was later shown to be an endoplasmic reticulum (ER) resident protein [4, 5]. HSP47 is a collagen-specific molecular chaperone that promotes the correct folding of type I and IV procollagen and prevents the aggregation of collagen molecules within the ER [6]. Under stress conditions, HSP47 expression is upregulated, and it acts as a molecular chaperone that mitigates cell damage from elevated temperature, heavy metals, and oxidative stress [7]. HSP47 has been identified as being overexpressed in various cancers, including cervical cancer [8], breast cancer [9], glioblastoma [10], and colorectal cancer [11]. In the case of breast cancer, HSP47 has been shown to facilitate cell invasion and metastasis by influencing the production of multiple extracellular matrix (ECM) proteins [9].

Despite its established importance in disease pathology and stress response, the detailed molecular mechanisms by which HSP47 contributes to the progression of GC remain largely unexplored. According to a recent study, HSP47 negatively correlates with E-cadherin (an epithelial marker) and positively with N-cadherin, MMP2, and MMP9 (markers associated with mesenchymal phenotype and invasion) and plays a critical role in GC progression through its regulation of epithelial-mesenchymal transition (EMT) and the Wnt/β-catenin signaling pathway [12]. The process of EMT is considered crucial in metastasis and is associated with the development of cancer [13]. However, the detailed molecular mechanisms of HSP47 in GC progression remain to be elucidated. Moreover, the role of HSP47 and its regulation in GC is not clear. This study aims to examine HSP47 expression in GC and to further explore possible carcinogenic mechanisms related to the prognosis of GC.

## Methods

### Cell lines and cell culture

The human GC cell line AGS was purchased from the American Type Culture Collection (ATCC) (Manassas, VA, USA), and other human GC cell lines were obtained from the Korean Cell Line Bank (KCLB) (Seoul, Korea). Cells were maintained in RPMI 1640 medium (Thermo Scientific HyClone, Logan, UT, USA) containing 10% fetal bovine serum (FBS) (Thermo Scientific HyClone) and 1% antibiotic solution (Gibco Invitrogen, Carlsbad, CA, USA). All human cell lines were incubated in a humidified atmosphere with 5% CO_2_ at 37 °C.

### Human tissues

Sixteen pairs of normal and GC tissues, used in quantitative real-time PCR (qRT-PCR) and Western blot analyses, were obtained through endoscopic biopsy from patients after obtaining their informed consent. The protocols were approved by the Institutional Review Board of the National Cancer Center, Korea (NCCNCS-08–127). Clinicopathological information of patient samples is summarized in Table 1. All patients provided written informed consent, which was approved by the Institutional Review Board.

**Table 1:** Features of Korean patients who provided GC tissues used in this study (*n* = 16)

			Number of patients (%)
Number of patients		Total	16
		Male	11 (68.7%)
		Female	5 (31.3%)
Age at diagnosis (years)		Range	34–74
		Mean ± SD	59.4 ± 12.5
Disease stage	T classification	T1	10 (62.5%)
		T2	2 (12.5%)
		T3	2 (12.5%)
		NA	2 (12.5%)
	N classification	N0	9 (56.3%)
		N1	3 (18.6%)
		N2	1 (6.3%)
		N3	1 (6.3%)
		NA	2 (12.5%)
	M classification	M0	14 (87.4%)
		M1	1 (6.3%)
		NA	1 (6.3%)
Lauren classification		Diffuse	7 (43.7%)
		Intestinal	5 (31.3%)
		Mixed	3 (18.7%)
		NA	1 (6.3%)

### Quantitative real-time PCR (qRT-PCR) and reverse transcription PCR

Total RNA purified using the illustra™ triplePrep Kit (GE Healthcare, Little Chalfont, UK) was reverse transcribed to cDNA using Superscript III reverse transcriptase (Invitrogen). This cDNA was then utilized for the relative quantification of HSP47 mRNA by qRT-PCR, normalized against glyceraldehyde 3-phosphate dehydrogenase (GAPDH). The primers used are listed in Supplementary Table 1. The QuantiFast SYBR Green PCR master mix (Qiagen, Hilden, Germany) was employed in a LightCycler 480 system (Roche Applied Science, Mannheim, Germany). RT-PCR was performed using Taq DNA polymerase (SolGent, Daejeon, Korea).

### Western blotting

The cell lysate was prepared by homogenizing cultured cells in PRO-PREP™ Protein Extraction Solution (iNtRON Biotechnology, Korea). Tissue lysates were prepared using 2-D DIGE buffer (7 M urea, 2 M thiourea, 4% CHAPS, and 20 mM Tris–HCl, pH 8.5). The lysates were then separated on 4–12% NuPAGE gels (Invitrogen) and transferred onto Immobilon-P membranes (Millipore, Billerica, MA, USA). Western blotting was conducted using anti-HSP47 (sc-8352, Santa Cruz Biotechnology, CA, USA), anti-beta-actin (A5441, Sigma-Aldrich, MO, USA), and anti-α-tubulin (T5168, Sigma-Aldrich) antibodies. Signal intensity was quantified using Multi Gauge V3.0 (Fujifilm, Tokyo, Japan), with background subtraction applied.

### siRNA transfection and cell proliferation assay

To silence HSP47 expression, 1.5 × 10^5^ AGS cells were transfected with small interfering RNA (siRNA) specific for human HSP47 (siRNA: 5′-CAAGAACUUAUUUGUACAUUU-3′) or with AccuTarget™ Negative control siRNA (non-targeting siRNA; Bioneer, Daejeon, Korea). Transfection was performed in a 6-well plate using HiPerFect (Qiagen) transfection reagent. The transfected cells were monitored by Western blotting every 24 h. Cell proliferation was assessed using the MTT assay. After transfection, the cells were cultured for 96 h and monitored at 24-h intervals.

### In vitro wound healing, migration, and invasion assays

For the wound-healing assays, siRNA-transfected AGS cells were cultured until they reached complete confluence. Subsequently, a scratch was made, and the percentage of the cell-free area was measured relative to the initial distance at 0 h (100%) using photographic images. In vitro migration and invasion assays were conducted using 8.0-μm pore inserts in a 24-well trans-well plate (Corning Incorporated, Corning, NY). Cells were seeded in serum-free RPMI 1640 medium in the upper chamber without coating for migration, or on filters coated with 10% Matrigel (BD Bioscience, San Jose, CA) for invasion, and placed in the lower chamber containing RPMI 1640 medium supplemented with 10% FBS. After 24 h of incubation, the migrating cells were stained with 1% crystal violet. The cells that had migrated to the lower surface of the filter were dissolved in 10% acetic acid, and the absorbance was measured at 564 nm (A_564_). After 48 h of incubation, cells that had invaded the lower chamber from the upper chamber were stained with a Diff-Quik staining kit (Sysmex, Kobe, Japan). The number of invading cells was counted in four microscopic fields per well at 20 × magnification. Each experiment was conducted in triplicate. The average number of cells was presented.

### Methylation-specific PCR (MSP) analysis and 5-aza-dC treatment

Genomic DNA (gDNA) samples extracted from the cell lines using the DNeasy kit (Qiagen), and from gastric tissues using the illustra™ triplePrep Kit (GE Healthcare), were treated with sodium bisulfite using the EZ DNA Methylation-Gold Kit (Zymo Research, Orange, CA). The bisulfite-modified gDNA was then amplified using HotStar Taq DNA polymerase and the primers described in a previous paper [14]. For demethylation, cells were seeded 24 h prior to treatment and incubated in media containing 20 µM 5-aza-2’-deoxycytidine (5-aza-dC; Sigma) for 3 days. Afterward, the medium was changed every 24 h, and the cells were harvested for further analysis.

### TCGA pan-cancer dataset

The normalized expression level of the *SERPINH1* gene and clinical information for 415 tumor and 35 normal samples were retrieved from TCGA pan-cancer (STAD) (https://xenabrowser.net/). Survival analysis was performed and plotted using R package survival v3.5.7 and survminer v0.4.9, respectively.

### Statistical analyses

The statistical significance of the data was determined using the Student *t*-test to estimate the difference between groups. Statistical significance was inferred at *P* < 0.05. The multivariate Cox proportional hazard model included disease stage, age, sex, and expression level of the *SERPINH1* gene.

## Results

### HSP47 expression is increased exclusively in human GC

**Fig. 1:**
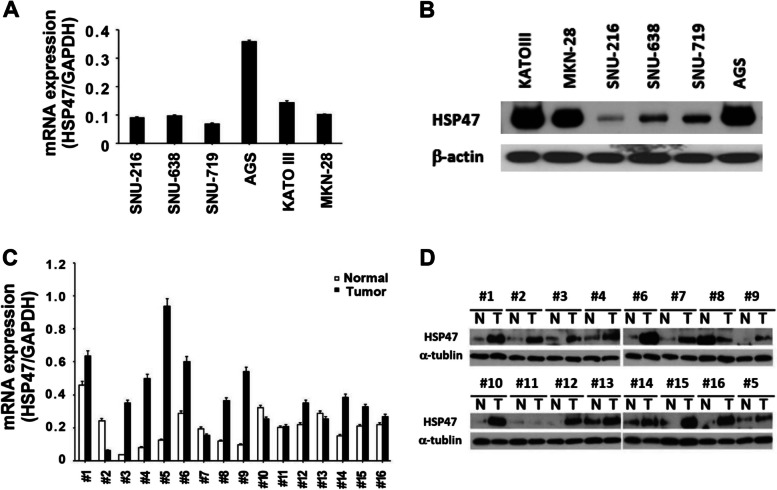
Upregulation of HSP47 in human GC cell lines and tissues. **A** HSP47 mRNA expression in six GC cell lines (SNU-216, SNU-638 SNU-719, AGS, KATO III, and MKN-28) detected by qRT-PCR. **B** Western blotting of HSP47 protein in the same GC cell lines. β-actin was used as a loading control. **C** HSP47 mRNA expression detected in 16 pairs of matched GC patient tissues. **D** Western blotting of HSP47 protein in the same 16 pairs of matched GC patient tissues. (N, adjacent noncancerous gastric tissue; T, gastric tumor tissue)

**Fig. 2:**
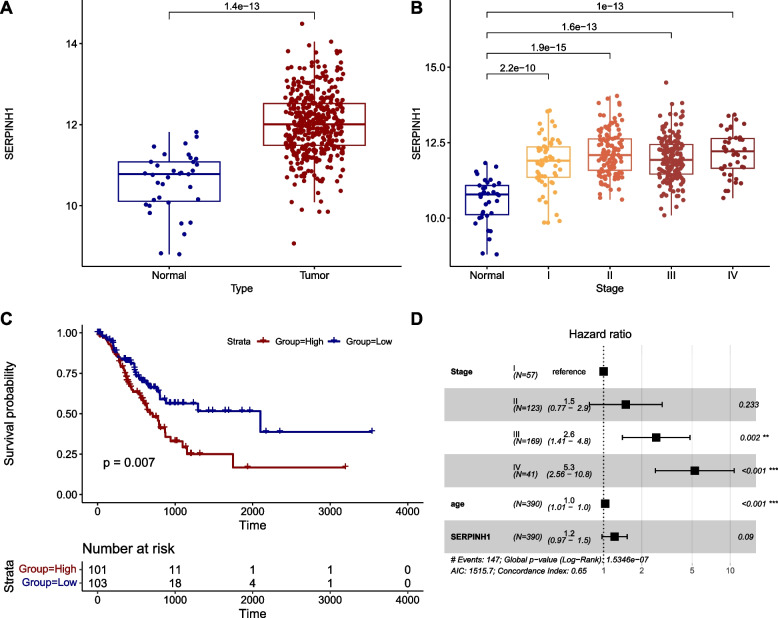
Expression level of the *SERPINH1* gene in the TCGA-STAD and its prognostic impact. **A** Relative expression of the *SERPINH1* gene between normal (*n* = 35) and tumor tissues (*n* = 415). **B** Relative expression of *SERPINH1* gene between disease stages. **C** Overall survival stratified by expression of *SERPINH1* gene. The top 25% of expression levels were classified as the High group and the bottom 25% as the Low group. **D** Multivariate Cox proportional hazard analysis with *SERPINH1* gene

From our previous gene expression profiling study of human gastric tumor and normal tissues, we identified human *HSP47* as an upregulated gene in tumor tissues (*P* < 0.0001). To validate this finding, we examined HSP47 expression by qRT-PCR and western blot analysis. HSP47 mRNA expression in the AGS GC cell line was higher than that in the other five GC cell lines (Fig. 1A). The levels of HSP47 protein expression in the AGS, KATO III, and MKN-28 GC cell lines are shown in Fig. 1B. Furthermore, HSP47 mRNA expression was dramatically upregulated in 12 of 16 (75%) Korean GC tissues, compared with its expression in the normal tissue counterparts (paired *t*-test: *P* = 0*.*008) (Fig. 1C). Similarly, HSP47 protein expression followed the mRNA expression pattern in the same set of GC tissues and their matched normal tissues. On average, HSP47 protein expression was increased by more than 2.3-fold in 14 out of 16 cancer tissues (87.5%), compared to the expression in the normal counterparts (*P* < 0.001) (Fig. 1D). This trend was further supported by TCGA pan-cancer data (STAD), revealing a significant upregulation in *SERPINH1* gene expression in tumors compared to normal tissues (*P* = 1.4e − 13) (Fig. 2A), even in early-stage cancer (stage I; *P* = 2.2e − 10) (Fig. 2B). These data strongly suggest that HSP47 expression is specifically elevated in GC. To address the functional significance of the association of HSP47 expression with clinical outcomes in GC, we performed Kaplan–Meier survival analysis using gene expression profiles and clinical data from TCGA-STAD. GC patients with high *SERPINH1* gene expression exhibited a significantly worse prognosis compared to those with low expression (*P* = 0.007) (Fig. 2C). However, Cox multivariate analysis revealed that expression level of *SERPINH1* gene was not an independent prognostic marker (*P* = 0.09) (Fig. 2D).

### Silencing HSP47 expression reduces the proliferation of human GC cells

**Fig. 3:**
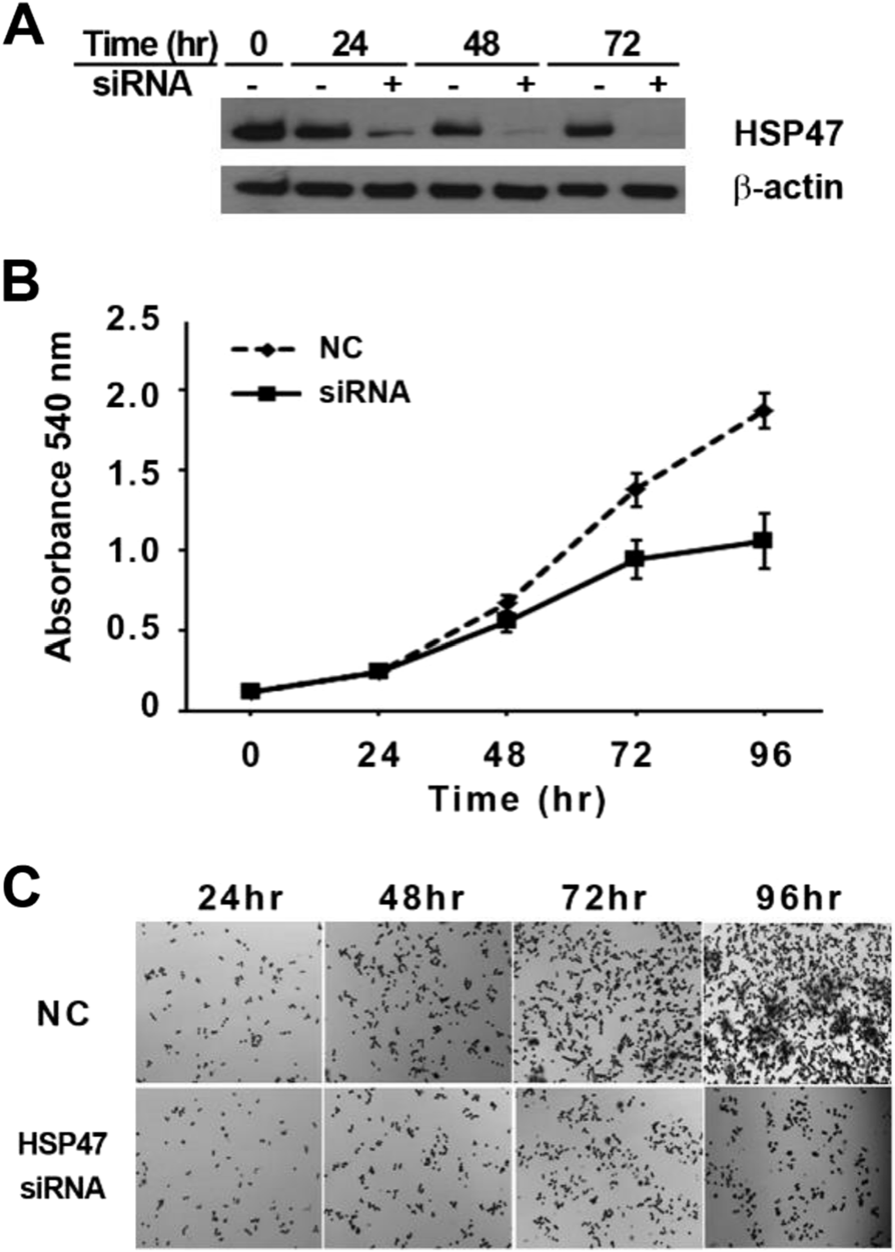
Effect of HSP47 on cell proliferation. **A** The inhibition of HSP47 expression by HSP47 siRNA transfection was confirmed by western blotting. HSP47 protein expression dramatically decreased from 24 h onward in AGS cells transfected with HSP47 siRNA. **B** MTT assay of siRNA-treated cells showing that HSP47 siRNA slowed the proliferation of AGS cells. **C** Proliferation patterns of AGS cells at 24-h intervals. The proliferation of HSP47 siRNA-transfected cells was retarded, unlike in non-targeted siRNA controls (NC); this pattern is similar to that of the cells shown in **B**

To understand the role of HSP47 in GC tumorigenesis, we examined the effect of HSP47 silencing on AGS human GC cells using siRNA. Silencing of HSP47 expression was confirmed by Western blotting, and the siRNAs used completely diminished the expression of HSP47. Expression of HSP47 was reduced within 24 h of transfection with HSP47-specific siRNA, whereas the non-targeting control siRNA (NC) had no effect on the level of HSP47 protein (Fig. 3A). The MTT assay revealed that the growth of HSP47-silenced cells slowed 48 h after transfection. After 96 h, cell proliferation was retarded by approximately 43% compared to that in control cells, which continued to grow rapidly (Fig. 3B). Microscopic imaging to observe proliferation patterns among the MTT assays indicated that cell proliferation was more significantly retarded in HSP47 siRNA-transfected cells than in control cells (Fig. 3C).

### HSP47 silencing inhibits the migration and invasion of human GC cells

**Fig. 4:**
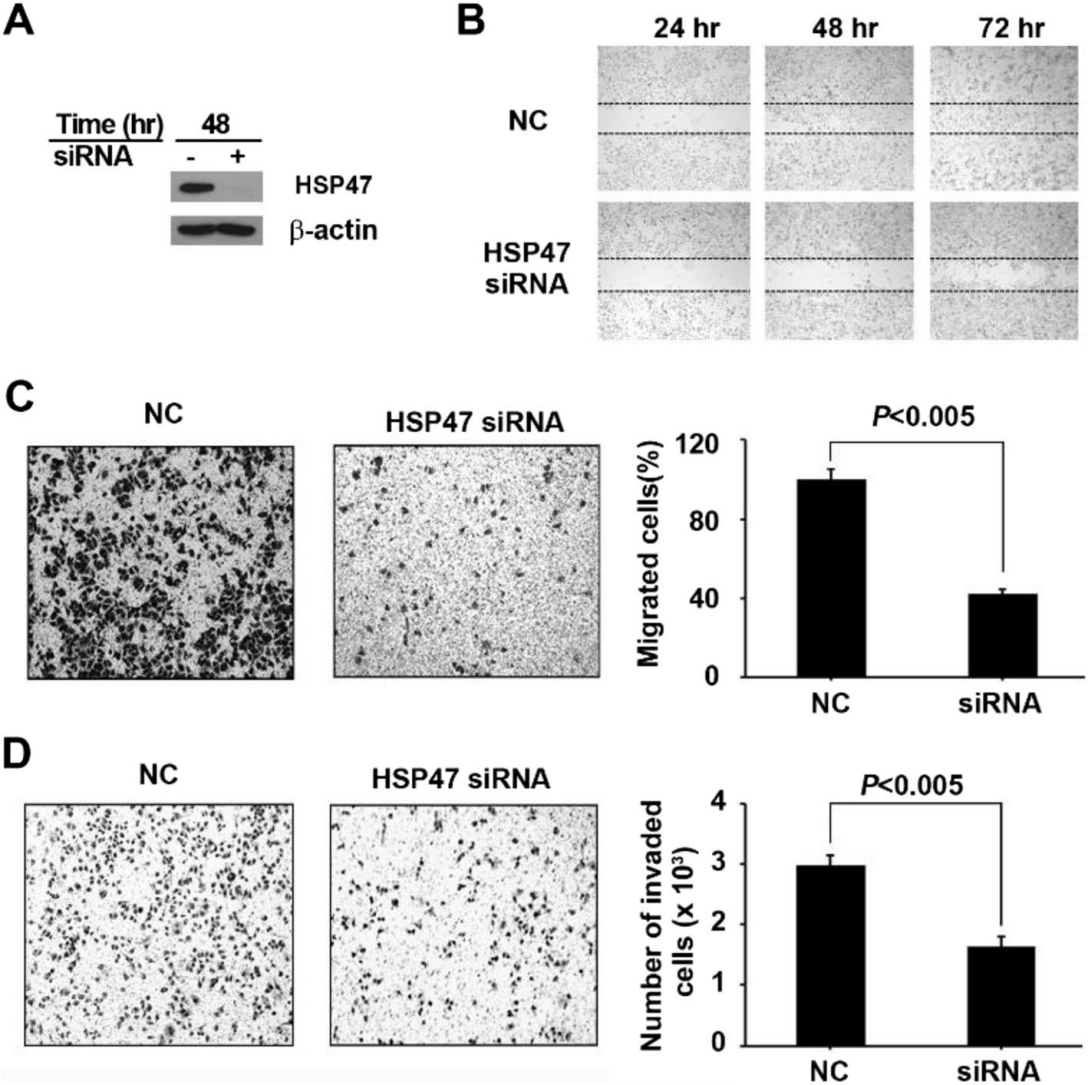
Effect of HSP47 silencing on GC wound-healing, cell migration, and invasion in vitro. **A** Western blot analysis shows the silencing of HSP47 protein expression in AGS cells by siRNA. **B** Wound-healing capability was considerably inhibited in HSP47 siRNA-transfected AGS cells. **C** The cell migration capability was inhibited by about 58%. The bar graph indicates the mean percentage of migrated cells at 24 h after incubation (NC = 100%, *P* < 0.005, *n* = 3). **D** HSP47 siRNA treatment dramatically inhibited the invasion of GC cells by approximately 45%. The bar graph shows the mean invaded cell number at 48 h after incubation (*P* < 0.005, *n* = 3)

Furthermore, we evaluated the in vitro wound-healing, migration, and invasion abilities after transfecting cells with HSP47 siRNA. Downregulation of HSP47 in cells transfected with HSP47 siRNA (Fig. 4A) decreased their wound-healing ability (Fig. 4B). Cell migration assays demonstrated that the number of migrated cells in HSP47 siRNA-transfected AGS cells significantly decreased by about 58% (*P* < 0.005) (Fig. 4C). Similarly, invasiveness was reduced by 44% in HSP47 siRNA-transfected AGS cells, compared to cells transfected with negative control siRNA (*P* < 0.005) (Fig. 4D). These results suggest that decreased expression of HSP47 reduces cell mobility and invasiveness in human gastric cells.

### Silencing of HSP47 inhibits matrix metallopeptidase-7 (MMP-7) expression

**Fig. 5:**
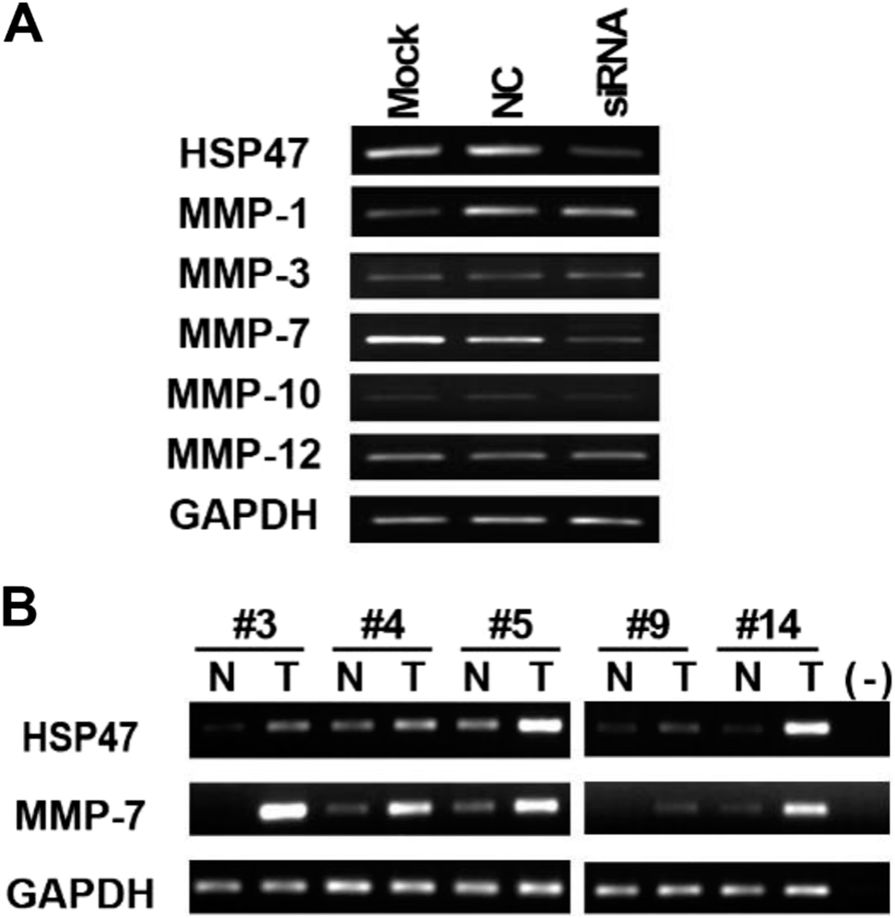
Effect of HSP47 silencing on the expression of matrix metalloproteinases (MMPs) genes detected by RT-PCR in AGS cells. **A** After HSP47 silencing, only MMP-7 mRNA expression was downregulated. **B** MMP-7 expression was upregulated in HSP47 highly-expressing GC tissues compared with the expression in paired normal gastric tissues. Five pairs of the non-cancerous and tumor tissues from Fig. 1 were used. GAPDH was used as a loading control

To investigate the mechanism by which HSP47 inhibits cell migration and invasion, we examined the effect of HSP47 suppression on well-documented cell migration- and invasion-related genes, such as matrix metallopeptidases (MMPs), in AGS cells. The mRNA expression of five MMP genes (MMP-1, MMP-3, MMP-7, MMP-10, and MMP-12) was detected by RT-PCR. Only MMP-7 expression was significantly reduced 72 h after transfection with HSP47 siRNA (Fig. 5A). MMP-2 and MMP-9, genes reportedly upregulated in other metastatic GCs [15, 16], were not detected in AGS cells. To investigate whether in vitro expression correlated with expression in vivo, we analyzed the mRNA expression of MMP-7 in gastric tumor samples that exhibited upregulated HSP47. As shown in Fig. 5B, MMP-7 mRNA expression was higher in tumor tissues with elevated levels of HSP47 than in non-cancerous tissues.

### Promoter DNA methylation regulates HSP47 expression

**Fig. 6:**
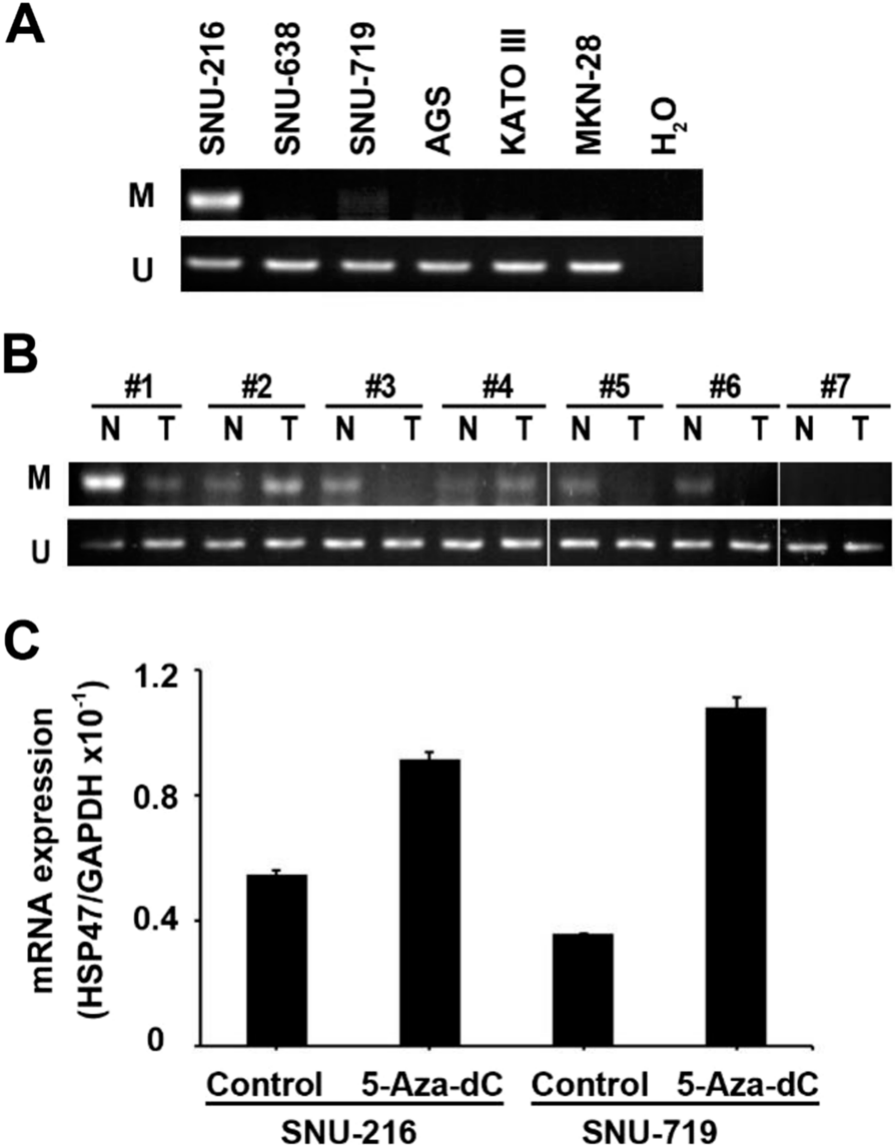
Methylation status of the HSP47 gene in GC cell lines and clinical tumors. **A** Methylation-specific PCR (MSP) analysis of the HSP47 promoter region between nucleotides − 138 and − 4 near the transcriptional start site in six GC cell lines (M, methylated; U, unmethylated). **B** MSP analysis of the HSP47 promoter region in clinical samples. Seven pairs of the non-cancerous and tumor tissues from Fig. 1 were used. **C** Expression levels of HSP47 mRNA before and after 5-aza-dC treatment in the low–HSP47-expressing cell lines SNU-216 and SNU-719

Yang et al. reported that the silencing of HSP47 in neuroblastoma cells was associated with the aberrant methylation of promoter CpG islands [14]. To investigate the mechanism of HSP47 expression in GC, we examined the methylation status of promoter CpG sites in six GC cell lines and seven pairs of gastric tissue samples. In the methylation-specific PCR (MSP) analysis, the methylated band was clearly amplified in the GC cell line SNU-216, which expressed low levels of HSP47 mRNA, while the unmethylated band was detected in all GC cells (Fig. 6A). Although, the unmethylated band was detected in both gastric normal and tumor tissues, in four of the seven clinical samples, the methylated band was observed only in the non-cancerous tissues (Fig. 6B). HSP47 mRNA and protein expression are shown in Fig. 1C and D, respectively. To validate the methylation-dependent expression of HSP47 in GC, we further analyzed the effect of the demethylating agent 5’-aza-2’-deoxycytidine (5-Aza-dC) on the low HSP47-expressing cell lines SNU-216 and SNU-719, as established in Fig. 1A. HSP47 expression increased in SNU-216 and SNU-719 GC cells treated with 5-Aza-dC, as evidenced by qRT-PCR analysis (1.7- and 3.0-fold increases, respectively) (Fig. 6C). These results suggest that DNA methylation suppresses HSP47 gene expression, while demethylation increases it in GC cell lines.

## Discussion

Multiple genes are associated with gastric tumorigenesis [17, 18]. In an effort to identify the genes responsible for the tumorigenesis of GC, we performed a genome-wide gene expression analysis of human GC and adjacent normal tissues from 27 Korean GC patients (NCBI GEO accession GSE30727) [19]. Recently, we demonstrated that interferon-induced transmembrane protein 1 (IFITM1) was upregulated in GC cell lines and human GC tissues, showing a correlation between its expression and the clinicopathologic features of patients with GC [2]. Here, we confirmed the expression of HSP47 in GC cell lines and tissues and examined the function of HSP47 in GC to enhance our understanding of the mechanisms involved in tumorigenesis.

In this study, we found that HSP47 mRNA and protein expression were highly upregulated in various GC cell lines and many human GC tissues. Similarly, previous studies have shown that HSP47 expression was upregulated in GC tissues [12, 20], and HSP47 was also detected in scirrhous GC by immunohistochemical analysis [21].

As a first step toward investigating whether the significant reduction of HSP47 expression impacted the biological behavior of GC cells, we silenced HSP47 in AGS cells with siRNA. We found that cell proliferation was slower in cells transfected with HSP47 siRNA than in control cells. Abnormal cell proliferation and cell cycle progression are hallmarks of aggressive malignant neoplasms, and upregulated protein synthesis is important to the etiology of cancer [22, 23]. Therefore, we suggest that elevated expression of HSP47 is an attribute of tumor tissues and/or proliferating cancer cell lines. This underscores the potential of targeting HSP47 in GC as a means to explore therapeutic interventions aimed at impeding tumor progression. For instance, approaches such as small molecule inhibitors and siRNA-based therapy targeting HSP47 could offer promising avenues for clinical treatment of gastric cancer.

The mechanistic insights gained from this study provide a clearer understanding of how HSP47 influences GC progression. Notably, the downregulation of HSP47 led to a significant decrease in matrix metallopeptidase-7 (MMP-7) expression, a gene closely associated with cancer cell migration and invasion. MMP-7 has a broad spectrum of proteolytic activity against a variety of ECM substrates, including type IV collagens, proteoglycans, laminin, fibronectin, and casein [24, 25]. As expected, MMP-7 expression was higher in human GC tissues than in normal tissues [26–28]. These findings suggest that MMP-7 expression has a tumorigenic effect on gastric cells and is related to HSP47 expression in GC. Additionally, the effect of promoter DNA methylation on HSP47 expression introduces an epigenetic dimension to its regulation, suggesting that alterations in the methylation status of the HSP47 promoter could be a potential mechanism by which GC cells modulate HSP47 expression to facilitate tumor progression.

To understand how HSP47 expression is regulated in GC cells, we examined the methylation status of the HSP47 promoter. HSP47 was found to be silenced and methylated in human neuroblastoma cell lines and tumors [14]. By contrast, our results showed that HSP47 was upregulated and un-methylated in most GC cell lines and tumor tissues. This suggests that HSP47 expression and promoter methylation status are cancer-specific alterations, although further studies on methylation in various cancers are needed.

Together, we identified a novel gene associated with GC and explored its potential role in tumorigenesis. Our findings clearly confirmed the involvement of HSP47 in GC progression, but further investigations are needed to elucidate the precise mechanisms underlying its function. Further, utilization of HSP47 in clinical settings will require more extensive research.

### Supplementary Information

Additional file 1. Table S1. List of primer sequences of used in this study.

Additional file 2. Full uncropped gel and blot image.

## Data Availability

No datasets were generated or analysed during the current study.
