# Supplementary material for: Overexpression of heat shock protein 47 is associated with increased proliferation and metastasis in gastric cancer
Source: Genomics Inform. 2024 Jun 17;22:6. doi: 10.1186/s44342-024-00010-7 (PMC11184955; doi:10.1186/s44342-024-00010-7)
Supplement: Supplementary file 1 — Additional file 1. Table S1. List of primer sequences of used in this study. [file 44342_2024_10_MOESM1_ESM.doc]

| Supplementary Table 1. List of primer sequences of used in this study. | |
| --- | --- |
|  |  |
| **Primer ID** | **Sequence (5' -> 3')** |
| HSP47-F | ACTAGTAGCCGCCCATAGCC |
| HSP47-R | GCACGTGCACTTTATTGAATG |
| GAPDH-F | TGCACCACCAACTGCTTA |
| GAPDH-R | GGATGCAGGGATGATGTTC |
| MMP1-F | CTGGAATTGGCCACAAAGTT |
| MMP1-R | CCTTCTTTGGACTCACACCA |
| MMP3-F | CCCTGGGTCTCTTTCACTCA |
| MMP3-R | TCAAAGGACAAAGCAGGATC |
| MMP7-F | CGGATGGTAGCAGTCTAGGG |
| MMP7-R | TGAATGGATGTTCTGCCTGA |
| MMP10-F | GGCTCTTTCACTCAGCCAAC |
| MMP10-R | TCCCGAAGGAACAGATTTTG |
| MMP12-F | CCTTCAGCCAGAAGAACCTG |
| MMP12-R | ACACATTTCGCCTCTCTGCT |
