# Supplementary material for: Overexpression of heat shock protein 47 is associated with increased proliferation and metastasis in gastric cancer
Source: Genomics Inform. 2024 Jun 17;22:6. doi: 10.1186/s44342-024-00010-7 (PMC11184955; doi:10.1186/s44342-024-00010-7)
Supplement: Supplementary file 2 — Additional file 2. Full uncropped gel and blot image. [file 44342_2024_10_MOESM2_ESM.pptx]

## Slide 1
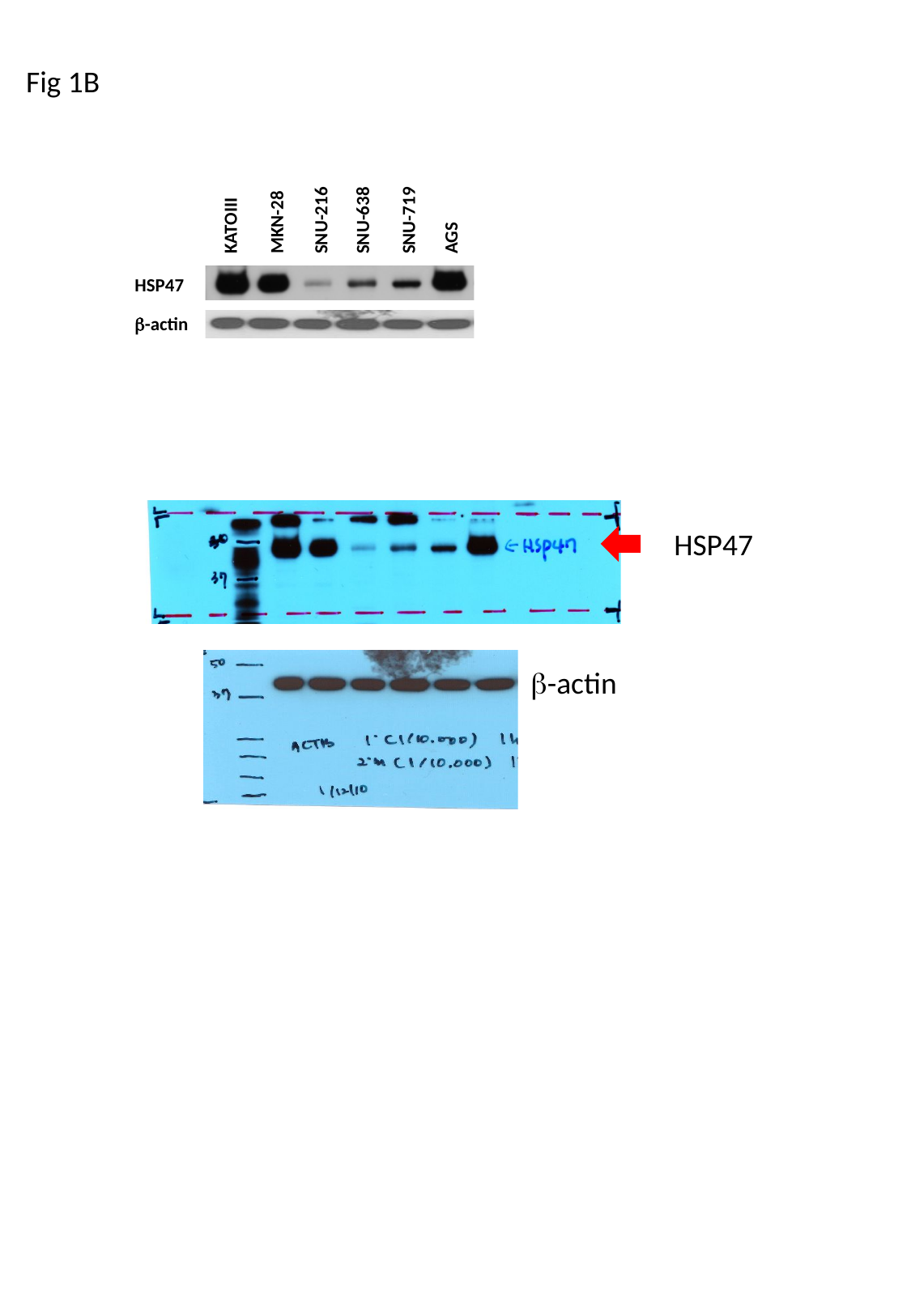

Fig 1B
KATOIII
MKN-28
SNU-638
SNU-719
AGS
SNU-216
HSP47
b-actin
HSP47
b-actin

## Slide 2
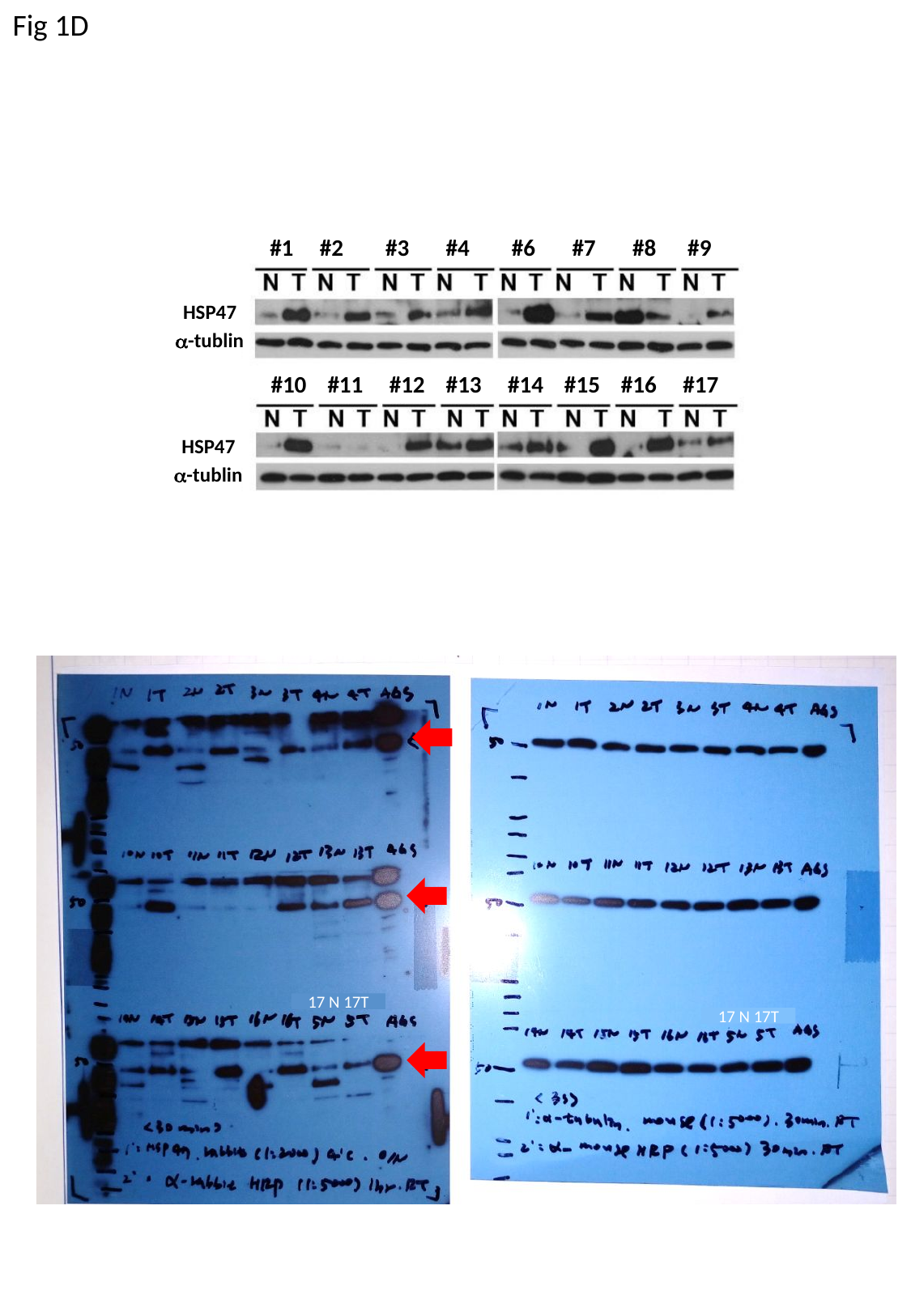

Fig 1D
#1 #2 #3 #4 #6 #7 #8 #9
HSP47
a-tublin
#10 #11 #12 #13 #14 #15 #16 #17
HSP47
a-tublin
17 N 17T
17 N 17T

## Slide 3
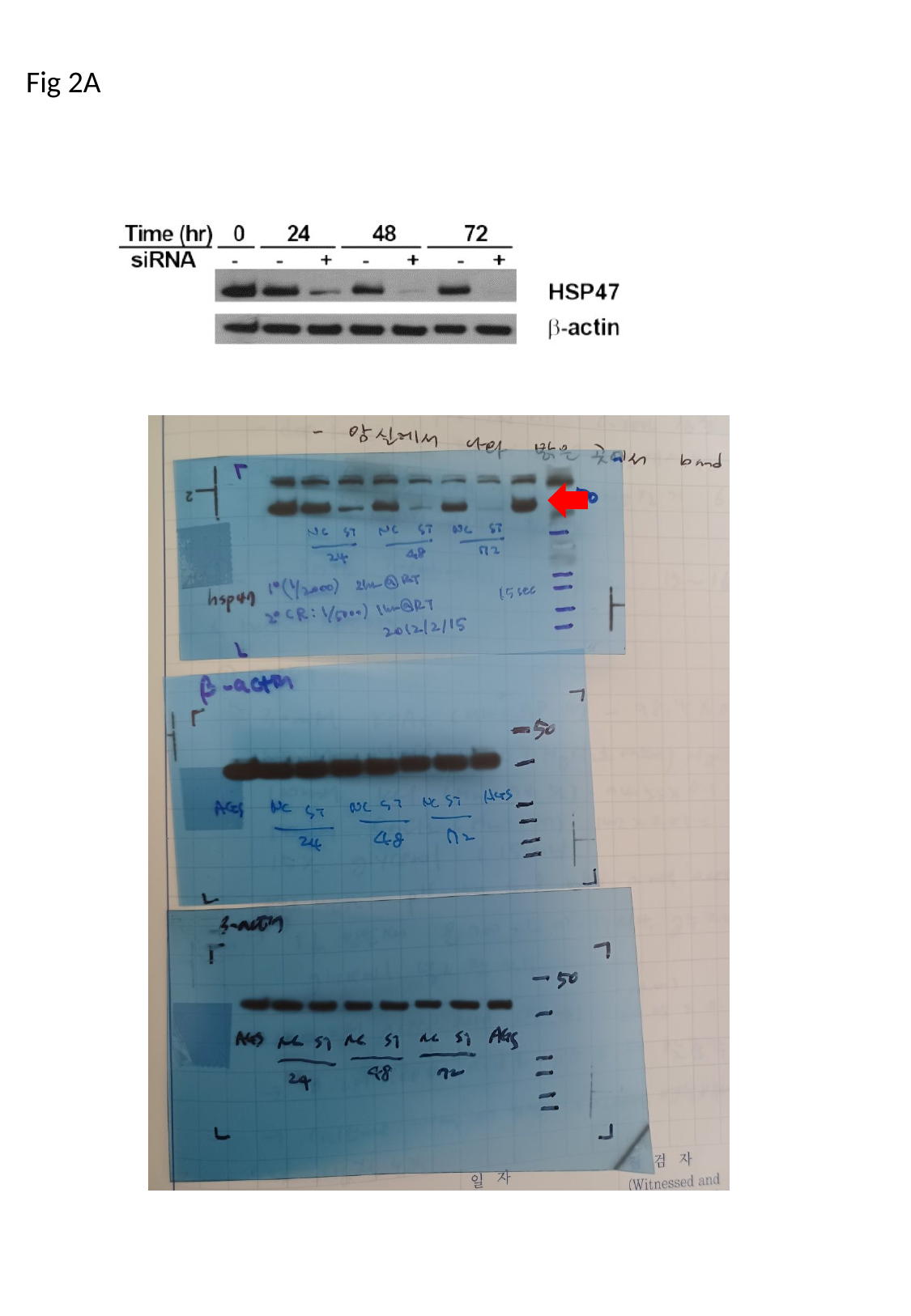

Fig 2A

## Slide 4
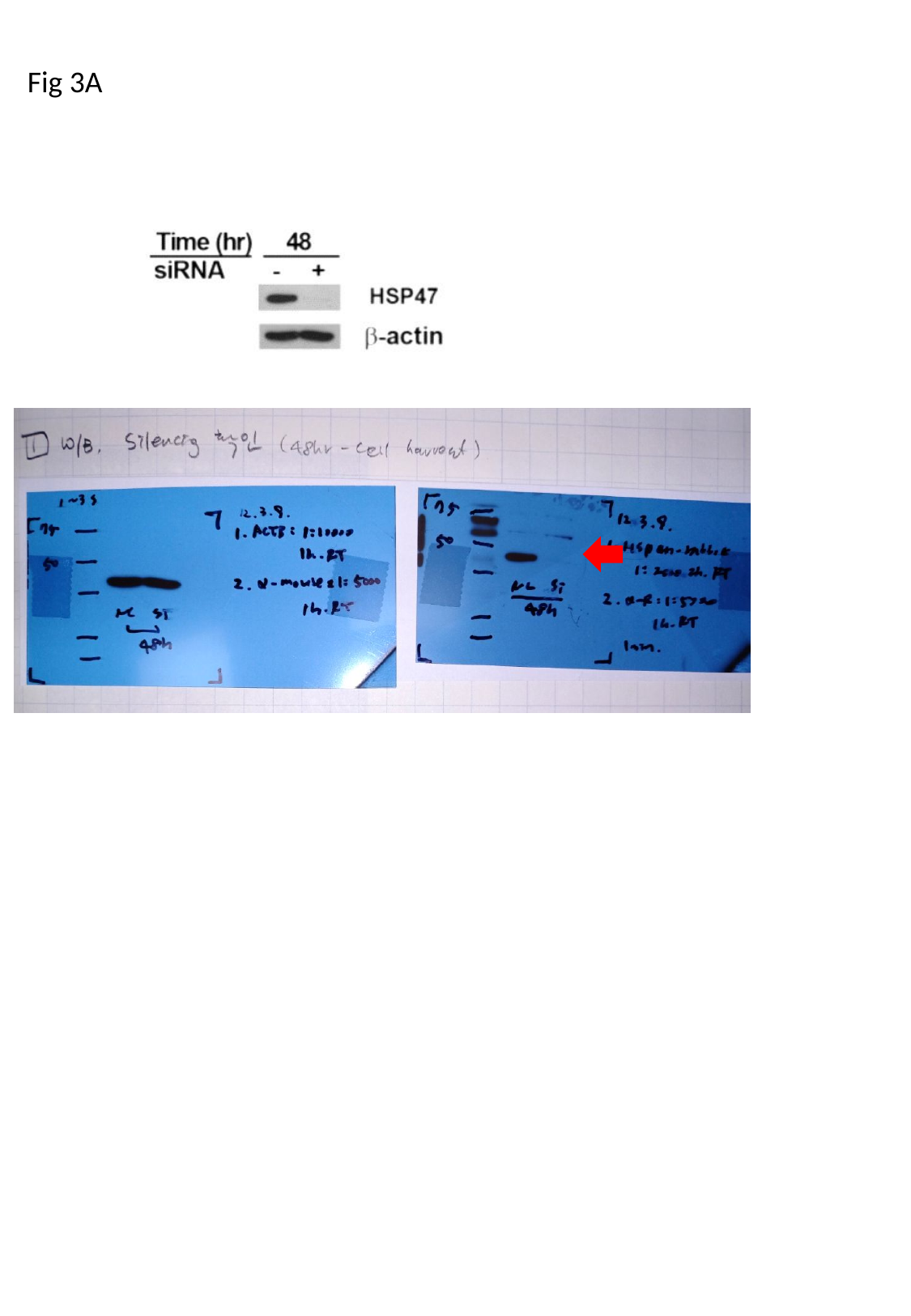

Fig 3A

## Slide 5
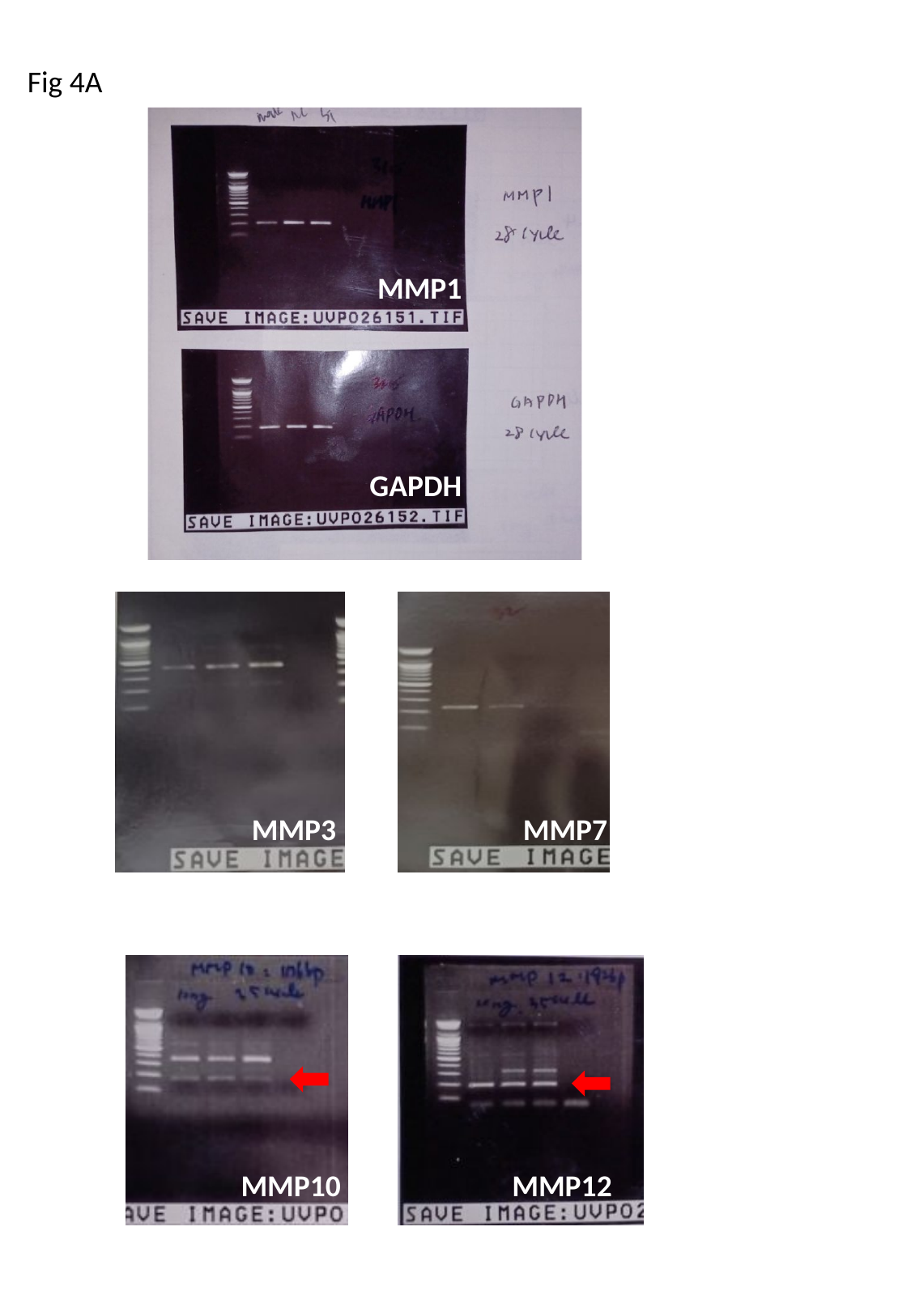

Fig 4A
MMP1
GAPDH
MMP3
MMP7
MMP10
MMP12

## Slide 6
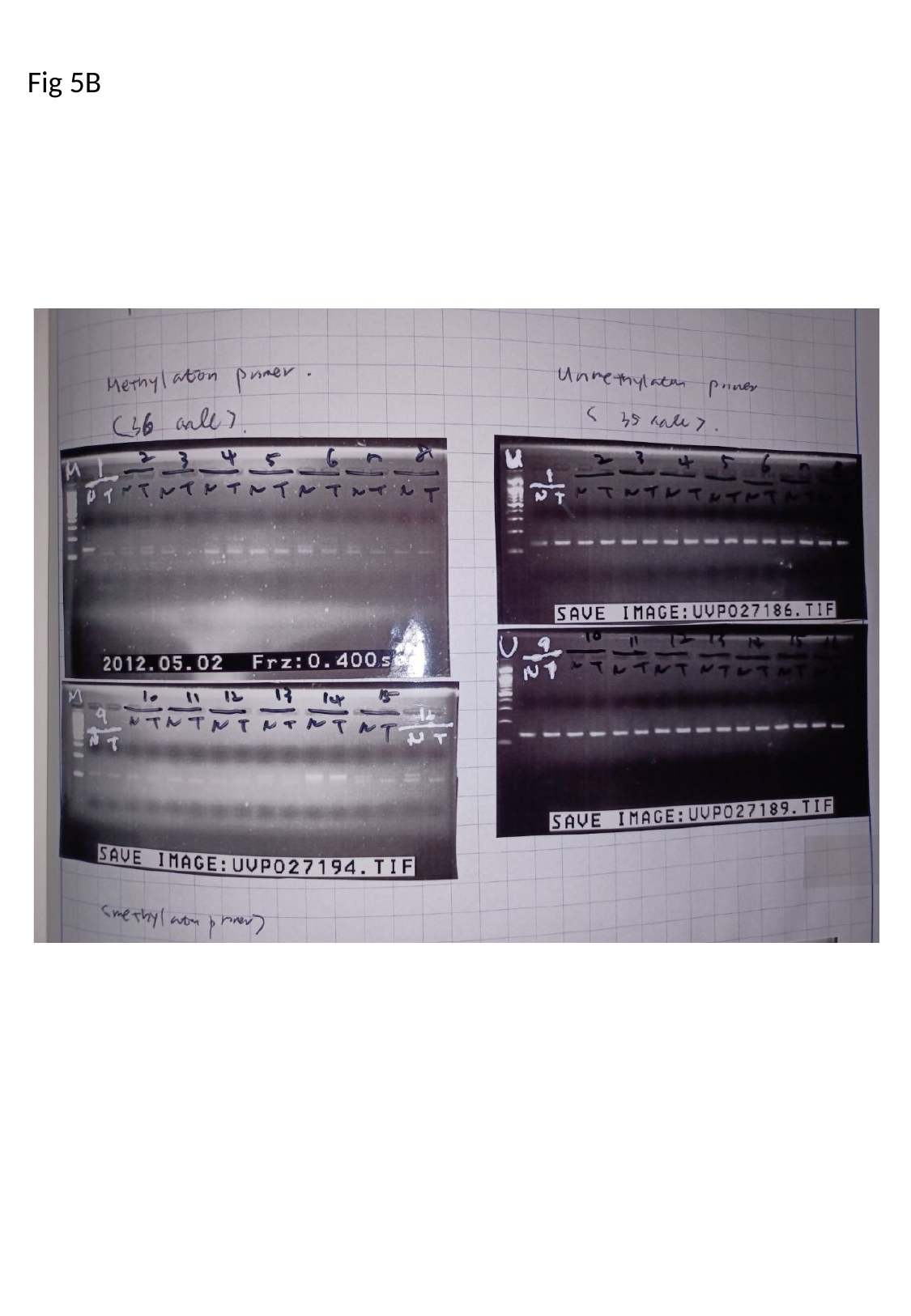

Fig 5B
